# Supplementary material for: Biodiversity across trophic levels drives multifunctionality in highly diverse forests
Source: Nat Commun. 2018 Jul 31;9:2989. doi: 10.1038/s41467-018-05421-z (PMC6068104; doi:10.1038/s41467-018-05421-z)
Supplement: Supplementary file 1 — Supplementary Information [file 41467_2018_5421_MOESM1_ESM.pdf]

SUPPLEMENTARY INFORMATION

Biodiversity across trophic levels drives multifunctionality in highly diverse forests

Schuldt et al.

Supplementary Table 1. Overview of the nine ecosystem functions and the 22 independent measurement variables used in the analyses of ecosystem multifunctionality

Ecosystem function	Measurement variables	Measurement details	Reference
E1. Erosion control	M1. Soil erosivity (inverse)	Estimated erosivity (calculated as the kinetic energy) of rain drops, measured with 15 sand-filled splash cups per plot in 2010.	8
E2. Microbial activity	M2. Acidophosphatase activity<br>M3. Xylosidase activity<br>M4. Beta-glucosidase activity<br>M5. N-acetyl-glucosaminidase activity	Enzyme assays of sieved, freeze-dried soil cores (4 cores per plot, upper 10 cm, September 2012), following standard methods[1]. Plates incubated at room temperature for 1 hour before measuring fluorescence (excitation wavelength 360 nm, emission wavelength 460 nm) with a 96-well plate reader (Synergy HT, Biotek). Enzyme activity calculated as nmol activity h^{-1} g dry soil equivalent^{-1}.	1
	M6. Fungal biomass<br>M7. Bacterial biomass	High-throughput variant of phospholipid fatty acid analysis[2,3] of the same soil (2 g subsample) sampled for enzyme analysis. Extracted three times with chloroform, methanol, and citrate buffer (1:1:0.9, by volume). Fatty acids contained in the lower chloroform phase saponified and converted into fatty acid methyl esters (strong acid (6N HCl) methylation). Extracted fatty acid methyl esters identified and quantified on an Agilent GCMS with a HP DB5 column. Peak areas converted to nmol lipid g^{-1} dry soil using known concentrations of internal standard (13:0 tridecanoic methyl ester). Soil microbial biomass calculated as the sum of all lipids ≤ 20 carbons in length.	9
E3. Nutrient cycling	M8. Ammonification rate<br>M9. Nitrification rate	In situ incubation[4,5] with PVC tubes (7 cm diameter, 25 cm length) carefully driven into the soil to sample intact cores consisting of litter layer and upper 10 cm of mineral soil. Tubes closed with a lid to prevent leaching of inorganic N to deeper soil layers. Aeration via two holes (5 mm diameter) in upper parts of tubes. By using in situ cores, plant uptake of inorganic N was avoided, soil structure was maintained and microbial activity continued under natural environmental conditions. Extractable ammonium and nitrate reanalyzed after an incubation period of 14 days.	

E4. Leaf decomposition	M10. Community leaf decomposition	Mean annual sum of leaf litter fall dry mass (measured 2009-2014, five 0.75 m x 0.75m litter traps with 1 mm nylon mesh, emptied monthly) divided by the mean leaf litter dry mass on the forest floor (determined with four sample cores in undisturbed litter patches in each plot and season).	
	M11. Leaf decomposition mixed species litterbags<br>M12. Leaf decomposition *Schima superba* litterbags	Litter bags (nylon, mesh width 1 mm) filled with mixed-species leaf litter (reflecting the composition of the dominant tree species of a given plot) and single-species (*Schima superba*), respectively. Replicated litter bags retrieved in six time steps between March 2009 and March 2010 to calculate the exponential decay coefficient *k*.	10
E5. Wood decomposition	M13. Community wood decomposition	Deadwood (lying and standing coarse woody debris of diameter > 10 cm, including dead trees) inventoried in all plots in 2009. Each deadwood piece labeled and re-inventoried in 2014. Three decay classes were used: undecayed solid wood with bark or bark starting to fall off (class I), partly decayed wood into which a knife can be pushed by hand (class II) and strongly decayed wood that can be easily fragmented by hand (class III). Estimation of deadwood biomass based on deadwood volume and species-specific wood density data for undecayed deadwood (class I). For classes II and III reduction in wood density (separately for broadleaved and conifer species) estimated based on wood samples obtained with drilling machine. Biomass of entire dead trees calculated by allometric equations using decay-class specific wood-density data[6]. Decomposition rates determined as the difference in biomass between the two inventories, assuming single exponential decay[7]	
	M14. Wood decomposition of *Schima superba*	Standardized wood samples (10 cm x 25 cm, including the bark) deposited with four spatial and two temporal replicates per plot. Temporal replicates retrieved one and two years after deposition, respectively. Mass loss of each sample recorded on a dry weight basis. Plot mean decomposition rates calculated as mass loss per time assuming single exponential decay[7]**.**	
E6. Primary productivity	M15. Basal area increment of woody plants > 10 cm DBH	Increment in stem basal area of woody plants from 2008 to 2012. Measurements of the diameter at breast height (DBH) were carried out on all	11

	M16. Basal area increment of woody plants > 3 and < 10 cm DBH	woody plants per plot with a DBH > 10 cm, as well as for all woody plants with a DBH > 3 cm and < 10 cm in the central 10 m x 10 m of each plot. Tree and shrub diameters were converted into stem cross-sectional area (basal area) and basal area increments were calculated, separately for woody plants > 10 cm DBH and woody plants < 10 cm DBH, as $m^2\ ha^{-1}\ year^{-1}$.	
E7. Herbivory resistance	M17. Canopy herbivory (inverse)	Measured from June to August 2010 on 20 trees per plot (four individuals of the five most abundant species per plot) with a DBH > 10 cm. Herbivory scored for all leaves of three branches collected from different heights of each tree. Leaves digitally scanned and leaf damage by insects visually estimated using damage classes of 0%, 1–5%, >5–10%, >10–25%, >25–50%, >50–75% and >75–100%.	12
	M18. Sapling herbivory (inverse)	Assessed in June/July 2008 for ten dominant woody plant species by visually estimating in the field the damage on all leaves of a maximum of ten saplings per species and plot. Damage classes defined as 0%, <1%, 1–5%, >5–15%, >15–35%, and >35% to account for the fact that low damage levels occurred much more frequently than high percentages of leaf damage.	13
E8. Predation	M19. Bait occurrence epigeic ants	Standardized cafeteria experiment conducted in the study plots in May 2012: tuna bait exposed on flat plastic dishes (two paired dishes at nine locations per plot) on the forest floor. All ants found feeding on the baits after 3 h were collected. Presence of ant species (i.e. their occurrence) counted per bait, summed occurrences per plot used as approximation of predation pressure by ants.	14
	M20. Predatory wasp broodcells with caterpillar prey<br>M21. Predatory wasp broodcells with spider prey	Standardized trap nests (reed internodes on two wooden posts with four traps each per plot) from September 2011 to October 2012. Internodes with Hymenoptera nests replaced by empty internodes on a monthly basis. For every nest, the number of overall and parasitized brood cells were counted. Number of brood cells that were provisioned with caterpillars (mostly brood cells of Vespidae: Eumeninae) and spiders (mostly cells of Pompilidae), respectively, used as measures of predation rate.	
E9. Parasitism	M22. Parasitized broodcells of cavity-nesting Hymenoptera	Standardized trap nests as described above. Parasitism quantified as number of parasitized brood cells divided by the overall number of brood cells	15

Supplementary Table 2. Biodiversity effects on multifunctionality as the average of nine standardized ecosystem functions

Predictor	Std. Est.	SE adj.	*z*	*P*	*N* mod.	Import.	Partial R^2
(Intercept)	0.33	0.05	6.3	< 0.001			
Woody plant composition (PC2)	0.23	0.06	3.8	< 0.001	2	1.0	0.52
Leaf chemical diversity (Rao's *Q*)	-0.17	0.05	3.7	< 0.001	2	1.0	0.41
Decomposer richness	0.15	0.04	3.5	< 0.001	2	1.0	0.45
Saprophytic fungi richness	-0.13	0.04	3.5	< 0.001	1	0.7	0.40
Trait composition (PC3)	-0.19	0.06	3.2	0.002	1	0.3	0.35

Multimodel averaging results (with a maximum of four predictors per individual model) of the effects of woody plant diversity and composition, and heterotrophic species richness on average multifunctionality. Mean adjusted R^2 across all models = 0.66. *N* mod. = Number of models in which the predictor was retained. Import. = Importance, sum of the Akaike weights of corresponding models. PC = principal component.

Supplementary Table 3. Biodiversity effects on multifunctionality as the number of functions surpassing a threshold percentage of the maximum observed value of each function

Predictor	Std. Est.	SE adj.	*z*	*P*	*N* mod.	Import.	Partial R^2
a) 30% threshold (mean R^2adj. = 0.67)							
(Intercept)	4.67	0.93	5.0	< 0.001			
Parasitoid richness	2.27	0.60	3.8	< 0.001	19	1.0	0.45
Woody plant composition (PC2)	2.67	0.99	2.7	0.007	17	0.9	0.30
Predator avg. richness	*1.16*	*0.73*	*1.6*	*0.113*	7	*0.4*	*0.13*
Woody plant species richness	*-1.26*	*0.77*	*1.6*	*0.101*	5	*0.3*	*0.13*
Stand age	*-1.06*	*0.63*	*1.7*	*0.093*	3	*0.2*	*0.13*
Woody plant density	*1.08*	*0.70*	*1.5*	*0.123*	2	*0.1*	*0.11*
Saprophytic fungi richness	*-1.04*	*0.65*	*1.6*	*0.108*	2	*0.1*	*0.12*
Leaf chemical diversity (Rao's Q*)*	*-1.34*	*0.86*	*1.6*	*0.118*	2	*0.1*	*0.12*
Herbivore avg. richness	*-1.26*	*0.76*	*1.7*	*0.096*	2	*0.1*	*0.16*
Environment (PC1)	-1.62	0.60	2.7	0.007	1	*0.0*	*0.26*
Trait composition (PC3)	*-1.13*	*0.84*	*1.4*	*0.175*	1	*0.0*	*0.90*
Environment (PC2)	*-1.19*	*0.90*	*1.3*	*0.186*	1	*0.0*	*0.80*
Leaf morphol. diversity (Rao's Q*)*	*1.18*	*0.80*	*1.5*	*0.137*	1	*0.0*	*0.11*
b) 60% threshold (mean R^2adj. = 0.54)							
(Intercept)	-0.07	2.18	0.0	0.973			
Environment (PC1)	4.61	1.07	4.3	< 0.001	1	0.7	0.50
Predator avg. richness	1.99	0.90	2.2	0.027	1	0.7	0.21
Leaf chemical diversity (Rao's *Q*)	-3.95	1.06	3.7	< 0.001	2	1.0	0.43
Woody plant composition (PC2)	4.88	1.81	2.7	0.007	2	1.0	0.36
Trait composition (PC3)	-3.26	1.27	2.6	0.010	1	0.3	0.26
Decomposer richness	4.04	0.98	4.1	< 0.001	1	0.3	0.48
c) 90% threshold (mean R^2adj. = 0.49)							
(Intercept)	2.13	0.46	4.6	< 0.001			
Leaf chemical diversity (Rao's *Q*)	-2.14	0.61	3.5	< 0.001	3	1.0	0.40
Saprophytic fungi richness	-1.35	0.50	2.7	0.007	3	1.0	0.29
Decomposer richness	1.25	0.52	2.4	0.017	3	1.0	0.26
Trait composition (PC3)	*-1.16*	*0.75*	*1.6*	*0.121*	*1*	*0.3*	*0.11*
Wood anatom. diversity (Rao's Q*)*	*0.73*	*0.48*	*1.5*	*0.128*	*1*	*0.3*	*0.11*

Multimodel averaging results (with a maximum of four predictors per individual model) of the effects of woody plant diversity and composition, and heterotrophic species richness on threshold-based multifunctionality. Non-significant (*P* > 0.05) predictors are printed in italics. *N* mod. = Number of models in which the predictor was retained. Import. = Importance, sum of the Akaike weights of corresponding models. PC = principal component.

Supplementary Table 4. Dimension reduction of woody plant species composition

**Woody plant species**	**PC2**
Quercus serrata Murray	-0.52
Eurya muricata Dunn	-0.22
Rhododendron simsii Planchon	-0.20
Rhododendron ovatum (Lindley) Planchon ex Maximowicz	-0.13
Rhododendron latoucheae Franchet	-0.11
Camellia fraterna Hance	-0.10
Camellia chekiangoleosa Hu	-0.09
…	…
Schima superba Gardner & Champion	0.10
Cyclobalanopsis glauca (Thunberg) Oersted	0.11
Adinandra millettii (Hooker & Arnott) Bentham & J. D. Hooker ex Hance	0.12
Vaccinium carlesii Dunn	0.16
Pinus massoniana Lambert	0.21
Lithocarpus glaber (Thunberg) Nakai	0.32
Loropetalum chinense (R. Brown) Oliver	0.58

Loadings of principal component 2 (woody plant composition PC2) selected from a principal components analysis (PCA) on woody plant species composition. Shown are the species with the lowest and highest values (i.e. the strongest loadings).

Supplementary Table 5. Path model results of biodiversity effects on average multifunctionality

**Model**					
Estimator	Maximum likelihood				
Number of observations	26				
RMSEA	0.0				
Chi-square	1.9				
Degrees of freedom	3				
P (Chi-square)	0.598				

Regressions

Response ~Predictor	Estimate	SE	*z*	*P*	Std. Estimate
Average multifunctionality~					
Decomposer richness	2.52	0.39	6.5	< 0.001	0.77
Saprophytic fungi richness	-1.35	0.34	-3.9	< 0.001	-0.39
Trait composition (PC3)	-1.83	0.50	-3.6	< 0.001	-0.40
Leaf chemical diversity (Rao's *Q*)	-2.01	0.40	-5.1	< 0.001	-0.44
Woody plant composition (PC2)	3.01	0.47	6.4	< 0.001	0.64
Decomposer diversity~					
Trait composition (PC3)	0.75	0.20	3.7	< 0.001	0.54
Leaf chemical diversity (Rao's Q*)*	*0.08*	*0.20*	*0.4*	*0.706*	*0.06*
Woody plant composition (PC2)	-0.54	0.21	-2.6	0.010	-0.38
Saprophytic fungi diversity~					
Decomposer richness	*0.14*	*0.22*	*0.6*	*0.516*	*0.15*
Trait composition (PC3)	*0.39*	*0.28*	*1.4*	*0.160*	*0.30*
Leaf chemical diversity (Rao's Q*)*	*0.03*	*0.23*	*0.1*	*0.897*	*0.02*
Woody plant composition (PC2)	*-0.32*	*0.26*	*-1.2*	*0.223*	*-0.23*

Variances

Variable	Estimate	SE	*z*	*P*	Std. Estimate
Average multifunctionality	0.18	0.05	3.6	< 0.001	0.20
Decomposer richness	0.05	0.01	3.6	< 0.001	0.56
Saprophytic fungi richness	0.06	0.02	3.6	< 0.001	0.76
Trait composition (PC3)	0.05	0.01	3.6	< 0.001	1.00
Leaf chemical diversity (Rao's *Q*)	0.05	0.01	3.6	< 0.001	1.00
Woody plant composition (PC2)	0.04	0.01	3.6	< 0.001	1.00

R^2

Variable	Estimate
Decomposer richness	0.44
Saprophytic fungi richness	0.24
Average multifunctionality	0.80

RMSEA = Root mean square error of approximation; PC = Principal component. Non-significant ($P > 0.05$) predictors are printed in italics.

Supplementary Table 6. Dimension reduction of community-weighted mean trait values (CWMs)

Trait	PC1	PC2	PC3
CWM leaf area	0.05	**0.56**	0.21
CWM specific leaf area	**-0.47**	0.28	-0.05
CWM leaf dry matter content	0.28	-0.10	**-0.52**
CWM leaf C content	0.28	0.05	**-0.47**
CWM leaf C:N ratio	**0.44**	-0.31	0.17
CWM leaf phenolics concentration	**0.44**	-0.10	0.36
CWM wood density	**-0.40**	-0.34	-0.15
CWM vessel diameter	0.15	0.29	**-0.52**
CWM fiber wall thickness	-0.21	**-0.53**	-0.10
Cumulative proportion explained	0.39	0.65	0.86

Loadings and eigenvalues of principal components (PC) selected from a principal components analysis (PCA) on the CWM of leaf and wood traits (most influential variables in bold).

Supplementary Table 7. Biodiversity effects across trophic levels on average multifunctionality (excluding functions directly mediated by a given trophic level)

**Predictor**	**Std. Est.**	**SE adj.**	***z***	***P***	***N* mod.**	**Import.**	**Partial *R^2***
a) Parasitoids (mean R^2adj. = 0.31) - without parasitism							
(Intercept)	0.39	0.04	10.3	< 0.001			
Parasitoid richness	0.09	0.04	2.3	0.024	2	1.0	0.21
Stand age	-0.11	0.05	2.2	0.028	2	1.0	0.20
Environment (PC1)	*0.05*	*0.04*	*1.0*	*0.300*	*1*	*0.3*	0.05
b) Predators (mean R^2adj. = 0.15) - without predation							
(Intercept)	0.45	0.03	16.1	< 0.001			
Stand age	*-0.11*	*0.05*	*-2.1*	*0.052*	*1*	*1.0*	*0.15*
c) Herbivores (mean R^2adj. = 0.22) - without herbivory resistance							
(Intercept)	0.42	0.03	15.4	< 0.001			
Herbivore avg. richness	-0.12	0.05	-2.6	0.016	1	1.0	0.22
d) Plants (mean R^2adj. = 0.50) - without primary productivity							
(Intercept)	0.18	0.08	2.3	0.022			
Woody plant composition (PC2)	0.37	0.09	4.2	< 0.001	4	1.0	0.49
Environment (PC1)	0.16	0.06	2.6	0.008	4	1.0	0.27
Leaf chemical diversity (Rao's *Q*)	-0.13	0.07	2.0	0.047	3	0.7	0.20
Stand age	*-0.10*	*0.05*	*1.9*	*0.058*	*2*	*0.5*	0.18
Trait composition (PC3)	*-0.07*	*0.06*	*1.1*	*0.258*	*1*	*0.1*	0.06
e) Decomposers (mean R^2adj. = 0.05) - without leaf and wood decomposition							
(Intercept)	0.40	0.06	7.1	< 0.001			
Stand age	*-0.16*	*0.14*	*1.2*	*0.249*	*2*	*0.5*	0.15
Decomposer richness x stand age	*0.41*	*0.22*	*1.8*	*0.066*	*1*	*0.2*	0.15
Decomposer richness	*-0.16*	*0.12*	*1.3*	*0.188*	*1*	*0.2*	0.08
Tree density	*0.04*	*0.06*	*0.7*	*0.477*	*1*	*0.1*	0.02
f) Fungi (mean R^2adj. = 0.22) - without decomposition, N-cycling, microbial activity							
(Intercept)	0.46	0.06	7.7	< 0.001			
Saprophytic fungi richness	-0.18	0.08	2.2	0.030	6	0.9	0.22
Stand age	*-0.14*	*0.08*	*1.7*	*0.093*	*3*	*0.5*	0.14
Pathogenic fungi richness	*0.13*	*0.09*	*1.5*	*0.137*	*3*	*0.4*	0.10
Tree density	*0.09*	*0.08*	*1.1*	*0.264*	*2*	*0.2*	0.06

Multimodel averaging results (with a maximum of four predictors per individual model) of the effects of woody plant diversity and composition, and heterotrophic species richness on threshold-based multifunctionality. Non-significant ($P > 0.05$) predictors are printed in italics. *N* mod. = Number of models in which the predictor was retained. Import. = Importance, sum of the Akaike weights of corresponding models. PC = principal component, avg = average.

Supplementary Table 8. Correlations (Pearson's *r*) among overall average multifunctionality and average multifunctionality indices excluding functions specific to individual trophic levels

	w/o predat.	w/o herbiv.	w/o product.	w/o decomp.	w/o decomp/ N/microb	All functions
Without Parasitism	0.88	0.86	0.90	0.81	0.72	0.93
Without predation		0.93	0.96	0.84	0.81	0.98
Without herbivory			0.91	0.81	0.75	0.95
Without primary productivity				0.86	0.77	0.97
Without decomposition					0.90	0.88
Without decomposition, N-cycling, microbial activity						0.84

All correlations were significant at $P < 0.001$.

Supplementary Table 9. Effects of average total community richness across all trophic levels, and across heterotrophs on multifunctionality as the average of nine standardized ecosystem functions

Predictor	Std. Est.	SE adj.	*z*	*P*	*N* mod.	Import.	Partial R^2
**Average total community richness**							
(Intercept)	-1.50	1.40	1.1	0.284			
Woody plant composition (PC2)	3.61	1.26	2.9	0.004	6	1.0	0.40
Environment (PC1)	1.62	0.74	2.2	0.029	5	0.7	0.20
Leaf chemical diversity (Rao's *Q*)	-1.97	0.81	2.5	0.014	4	0.6	0.25
Stand age	*-3.10*	*2.16*	*1.4*	*0.152*	*3*	*0.5*	*0.24*
Total avg. community richness	*-1.81*	*1.47*	*1.2*	*0.218*	*1*	*0.3*	*0.08*
Total community richness x stand age	6.27	2.73	2.3	0.021	1	0.3	0.22
Trait composition (PC3)	*-1.00*	*0.75*	*1.3*	*0.182*	*1*	*0.2*	*0.09*
Leaf morphol. diversity (Rao's Q)	*0.75*	*0.68*	*1.1*	*0.272*	*1*	*0.1*	*0.06*
**Average heterotrophic community richness**							
(Intercept)	-1.50	1.40	1.1	0.284			
Woody plant composition (PC2)	0.27	0.10	2.7	0.008	4	1.0	0.40
Environment (PC1)	0.13	0.06	2.2	0.025	3	0.6	0.22
Leaf chemical diversity (Rao's *Q*)	-0.16	0.06	2.4	0.015	3	0.6	0.25
Stand age	*-0.30*	*0.18*	*1.6*	*0.100*	*2*	*0.5*	*0.22*
Total avg. community richness (heterotrophs)	*-0.11*	*0.11*	*1.0*	*0.299*	*1*	*0.4*	*0.05*
Total community richness x stand age	0.43	0.20	2.2	0.030	1	0.4	0.20
Trait composition (PC3)	*-0.08*	*0.06*	*1.3*	*0.182*	*1*	*0.2*	*0.09*

Multimodel averaging results (with a maximum of four predictors per individual model) of the effects of woody plant diversity and composition, and a) average total community richness and b) heterotrophic average community richness on average multifunctionality. Mean adjusted R^2 across all models = 0.47 (total community richness) and 0.49 (heterotrophic community richness). Non-significant ($P > 0.05$) predictors are printed in italics. *N* mod. = Number of models in which the predictor was retained. Import. = Importance, sum of the Akaike weights of corresponding models. PC = principal component, avg = average.

Supplementary Table 10. Biodiversity effects on individual functions

**Predictor**	**Std. Est.**	**SE adj.**	***z***	***P***	***N* mod.**	**Import.**	**Partial R^2**
a) Erosion control (mean R^2adj. = 0.43)							
(Intercept)	-0.20	0.24	0.8	0.410			
Woody plant composition (PC2)	0.84	0.27	3.1	0.002	2	1.0	0.33
Environment (PC2)	-0.72	0.20	3.7	0.000	2	1.0	0.42
Environment (PC1)	0.57	0.17	3.3	0.001	2	1.0	0.36
Pathogenic fungi richness	*0.25*	*0.13*	*1.9*	*0.058*	*1*	*0.6*	*0.16*
b) Microbial activity (mean R^2adj. = 0.48)							
(Intercept)	0.29	0.10	2.9	0.004			
Environment (PC1)	0.41	0.14	2.8	0.004	5	1.0	0.39
Mycorrhizal fungi avg. richness	-0.34	0.14	2.5	0.014	5	1.0	0.25
Environment (PC2)	0.37	0.15	2.4	0.015	4	0.7	0.25
Leaf morphol. diversity (Rao's Q)	*-0.33*	*0.17*	*1.9*	*0.056*	*2*	*0.4*	*0.20*
Wood anatom. diversity (Rao's *Q*)	0.32	0.13	2.4	0.015	1	0.3	0.24
Predator avg. richness	*-0.18*	*0.13*	*1.4*	*0.169*	*1*	*0.2*	*0.09*
Pathogenic fungi richness	*-0.17*	*0.12*	*1.4*	*0.164*	*1*	*0.2*	*0.09*
c) N cycling (mean R^2adj. = 0.39)							
(Intercept)	0.13	0.09	1.4	0.162			
Mycorrhizal fungi avg. richness	0.42	0.18	2.4	0.018	6	1.0	0.25
Saprophytic fungi richness	-0.29	0.14	2.1	0.035	5	0.9	0.20
Trait composition (PC3)	*0.30*	*0.17*	*1.8*	*0.078*	*5*	*0.7*	*0.15*
Stand age	*0.24*	*0.12*	*2.0*	*0.050*	*2*	*0.5*	*0.17*
Tree density	*-0.17*	*0.11*	*1.6*	*0.112*	*1*	*0.2*	*0.12*
Herbivore avg. richness	*0.18*	*0.12*	*1.5*	*0.121*	*1*	*0.1*	*0.11*
d) Leaf decomposition (mean R^2adj. = 0.57)							
(Intercept)	0.44	0.06	7.7	< 0.001			
Decomposer richness	0.41	0.11	3.8	0.001	1	1.0	0.41
Environment (PC1)	-0.40	0.10	-4.2	< 0.001	1	1.0	0.45
Stand age	-0.35	0.09	-3.9	< 0.001	1	1.0	0.42
Woody plant species richness	0.24	0.10	2.4	0.028	1	1.0	0.21
e) Wood decomposition (mean R^2adj. = 0.53)							
(Intercept)	0.62	0.108	5.8	< 0.001			
Wood anatom. diversity (Rao's *Q*)	0.64	0.1434	4.4	< 0.001	2	1.0	0.50
Leaf morphol. diversity (Rao's *Q*)	-0.60	0.1981	3.0	0.002	2	1.0	0.34
Stand age	-0.59	0.1698	3.5	< 0.001	2	1.0	0.42
Decomposer richness	0.28	0.1357	2.1	0.038	1	0.7	0.19
f) Primary productivity (mean R^2adj. = 0.63)							
(Intercept)	0.22	0.0807	2.7	0.007			
Leaf chemical diversity (Rao's *Q*)	-0.45	0.107	4.2	< 0.001	3	1.0	0.50
Predator avg. richness	0.45	0.0916	4.9	< 0.001	3	1.0	0.57
Decomposer richness	0.24	0.0923	2.6	0.009	3	1.0	0.29
Trait composition (PC3)	*-0.24*	*0.126*	*1.9*	*0.056*	*1*	*0.5*	*0.16*
Saprophytic fungi richness	*-0.13*	*0.086*	*1.6*	*0.118*	*1*	*0.2*	*0.12*

g) Herbivory resistance (mean R^2adj. = 0.52)							
(Intercept)	0.95	0.1366	6.9	< 0.001			
Woody plant species richness	-0.39	0.1501	2.6	0.010	9	1.0	0.28
Trait composition (PC3)	*-0.32*	*0.173*	*1.8*	*0.069*	*5*	*0.6*	*0.16*
Saprophytic fungi richness	*-0.23*	*0.126*	*1.8*	*0.072*	*5*	*0.6*	*0.15*
Leaf chemical diversity (Rao's *Q*)	-0.36	0.1595	2.3	0.024	3	0.5	0.21
Stand age	-0.31	0.1375	2.2	0.026	3	0.3	0.21
Tree density	0.24	0.1217	2.0	0.044	3	0.3	0.17
h) Predation (mean R^2adj. = 0.57)							
(Intercept)	0.22	0.07	3.4	0.002			
Stand age	-0.41	0.10	-4.2	< 0.001	1	1.0	0.46
Decomposer richness	0.27	0.07	3.7	0.001	1	1.0	0.40
Trait composition (PC1)	0.20	0.09	2.4	0.027	1	1.0	0.21
Parasitoid richness	0.18	0.07	2.7	0.013	1	1.0	0.26
i) Parasitism (mean R^2adj. = 0.52)							
(Intercept)	0.24	0.316	0.8	0.449			
Mycorrhizal fungi avg. richness	-0.39	0.1793	2.2	0.030	6	0.8	0.23
Parasitoid richness	0.29	0.1404	2.1	0.038	6	0.8	0.23
Woody plant composition (PC2)	0.64	0.2633	2.4	0.015	5	0.6	0.33
Tree density	-0.35	0.1572	2.2	0.027	4	0.6	0.25
Wood anatom. diversity (Rao's *Q*)	0.28	0.1404	2.0	0.045	1	0.2	0.18
Herbivore avg. richness	*-0.24*	*0.145*	*1.6*	*0.099*	*1*	*0.1*	*0.13*

Multimodel averaging results (with a maximum of four predictors per individual model) of the effects of woody plant diversity and composition, and heterotrophic species richness on threshold-based multifunctionality. Non-significant ($P > 0.05$) predictors are printed in italics. *N* mod. = Number of models in which predictor was retained. Import. = Importance, sum of the Akaike weights of corresponding models. PC = principal component, avg = average.

Supplementary Table 11. Overview of previous analyses at the study site of relevance for a multidiversity-multifunctionality framework

Topic	Taxon/Function	Reference	Innovation
Environmental effects on functional groups of organisms	Multitrophic	16	Our study looks at environmental effects in the context of ecosystem multifunctionality
Effects of woody plant diversity and/or woody plant composition on the diversity and/or composition of individual heterotrophic taxa	Soil microorganisms	17,18	Unlike these previous studies, our study analyzes higher trophic level diversity as a predictor (effects of heterotrophic diversity on ecosystem (multi)functionality), rather than as a response variable, and it considers a much more comprehensive set of taxa than previously analyzed
	Herbivores, predators, decomposers	19,20	
	Spiders	21,22,23	
	Ants	24,25	
	Spider-ant interactions	14	
Biodiversity relationships among taxa	Multitrophic	26	Our study analyzes multitrophic diversity effects on ecosystem multifunctionality, whereas these previous studies were limited to biodiversity relationships among different taxa
	Multitrophic	27	
Effects of woody plant diversity and/or woody plant composition on individual ecosystem functions	Erosion	8	Our study considers additional functions and analyzes them in a much more comprehensive, multifunctional context. Moreover, it not only looks at potential effects of woody plants (as these previous studies), but at how higher trophic levels contribute to multifunctionality and potentially mediate the effects of woody plants
	Microbial activity and biomass (only 12 of 27 plots)	28	
	Leaf decomposition	10,29	
	Primary productivity	11,30	
	Herbivory	12,13,31	
	Parasitism	15	
Effects of higher trophic levels on individual ecosystem functions	Soil microbial diversity effects on wood decomposition	32	Our study considers simultaneous effects of a much larger number of organism groups on a wide range of ecosystem functions

Studies of individual functions or heterotrophic taxa and their relationship with woody plant communities previously conducted at the study site, and the innovation provided by the current study.

Supplementary Table 12. Mean and range of observed values of the ecosystem function and species richness measurements used in the analyses of ecosystem multifunctionality

Function/Trophic level	Measurement variable[1]	Mean	SD	Min.	Max.
E1. Erosion control	M1. Soil erosivity (multiplied by -1)	-859.36	193.65	-1405.03	-530.27
E2. Microbial activity	M2. Acidophosphatase activity	1304.87	302.31	866.92	2278.74
	M3. Xylosidase activity	43.16	20.84	18.02	95.17
	M4. Betaglucosidase activity	138.73	60.58	53.77	303.66
	M5. N-acetyl-glucosaminidase activity	202.41	102.27	51.40	536.44
	M6. Fungal biomass	22.13	9.14	9.38	42.83
	M7. Bacterial biomass	61.53	27.05	18.61	121.93
E3. Nutrient cycling	M8. Ammonification rate	0.34	0.24	-0.21	0.83
	M9. Nitrification rate	0.06	0.09	-0.003	0.31
E4. Leaf decomposition	M10. Community leaf decomposition	0.67	0.12	0.43	0.96
	M11. Leaf decomposition mixed species litterbags	0.51	0.11	0.35	0.86
	M12. Leaf decomposition *Schima superba* litterbags	0.45	0.09	0.32	0.67
E5. Wood decomposition	M13. Community wood decomposition	0.19	0.12	0.03	0.47
	M14. Wood decomposition of *Schima superba*	0.20	0.05	0.10	0.28
E6. Primary productivity	M15. Basal area increment of woody plants > 10 cm DBH	0.012	0.005	0.006	0.028
	M16. Basal area increment of woody plants > 3 and < 10 cm DBH	0.002	0.001	0.001	0.005
E7. Herbivory resistance	M17. Canopy herbivory (multiplied by -1)	-7.83	1.58	-11.75	-5.03
	M18. Sapling herbivory (multiplied by -1)	-7.55	2.12	-11.14	-4.13
E8. Predation	M19. Bait occurrence epigeic ants	11.15	2.65	8	17
	M20. Predatory wasp broodcells with caterpillar prey	70.42	37.30	8	117
	M21. Predatory wasp broodcells with spider prey	15.04	19.85	0	87

E9. Parasitism	M22. Parasitized broodcells of cavity-nesting Hymenoptera	0.11	0.08	0.007	0.32
T1. Parasitoids	S1. Parasitoid SR	4.1	2.4	1	8
T2. Predators	S2. Spider SR	45.6	6.3	28	58
	S3. Predatory ant SR	8.5	2.5	4	13
	S4. Omnivore ant SR	19.4	2.6	14	25
	S5. Chilopod SR	5.8	2.1	3	11
	S6. Wasp SR	3.1	1.2	1	5
T3. Primary consumers/herbivores	S7. Weevil SR	6.6	3.0	2	12
	S8. Bark beetle SR	20.7	5.7	10	31
	S9. Lepidoptera SR	11.0	3.4	5	20
	S10. Longhorn beetle SR	8.2	4.8	1	23
T4. Plants	S11. Woody plant SR	41.7	10.5	25	69
T5. Macrofaunal decomposers	S12. Decomposer SR	4.4	2.2	1	8
T6. Mycorrhizae	S13. Arbuscular mycorrhizae SR	9.5	3.8	3	18
	S14. Ectomycorrhizae SR	97.2	10.6	76	117
T7. Saprohytic fungi	S15. Saprophytic fungi SR	208.7	22.3	167	244
T8. Pathogenic fungi	S16. Pathogenic fungi SR	20.1	4.4	11	27

[1]See Supplementary Table 1 on units and details of measurements of the individual variables.
Mean, Min. and Max. are mean, minimum and maximum values observed at the plot level, SD = standard deviation. E = ecosystem function, M = measurement of ecosystem function, T = trophic level, S = species group, SR = species richness.
Note that observed values are based on the taxa considered for a given functional group and might represent an underestimate of the total species richness of this group (e.g. macrofaunal decomposers, parasitoids, arbuscular mycorrhizal fungi) due to limitations of taxonomic scope, sampling time, or primers used in the molecular methods. Nevertheless, these limitations apply equally for all study plots, making data relevant and comparable among plots.

Supplementary Table 13. Dimension reduction of environmental plot conditions

**Environmental variable**	**PC1**	**PC2**
Elevation	**0.41**	-0.12
Slope	0.04	**0.39**
Northness	0.15	0.14
Eastness	-0.12	0.04
Latitude	**0.27**	**-0.26**
Longitude	0.13	**0.32**
Soil pH	-0.22	-0.22
Soil N-content	**0.29**	**0.42**
Soil C-content	**0.27**	**0.49**
Soil C:N-ratio	-0.13	**0.26**
Mean January temperature	**-0.42**	0.14
Mean July temperature	**-0.37**	0.24
Mean annual temperature	**-0.41**	0.2
Cumulative proportion explained	0.38	0.57

Loadings and eigenvalues of principal components (PC) selected from a principal components analysis (PCA) on environmental variables (most influential variables in bold).

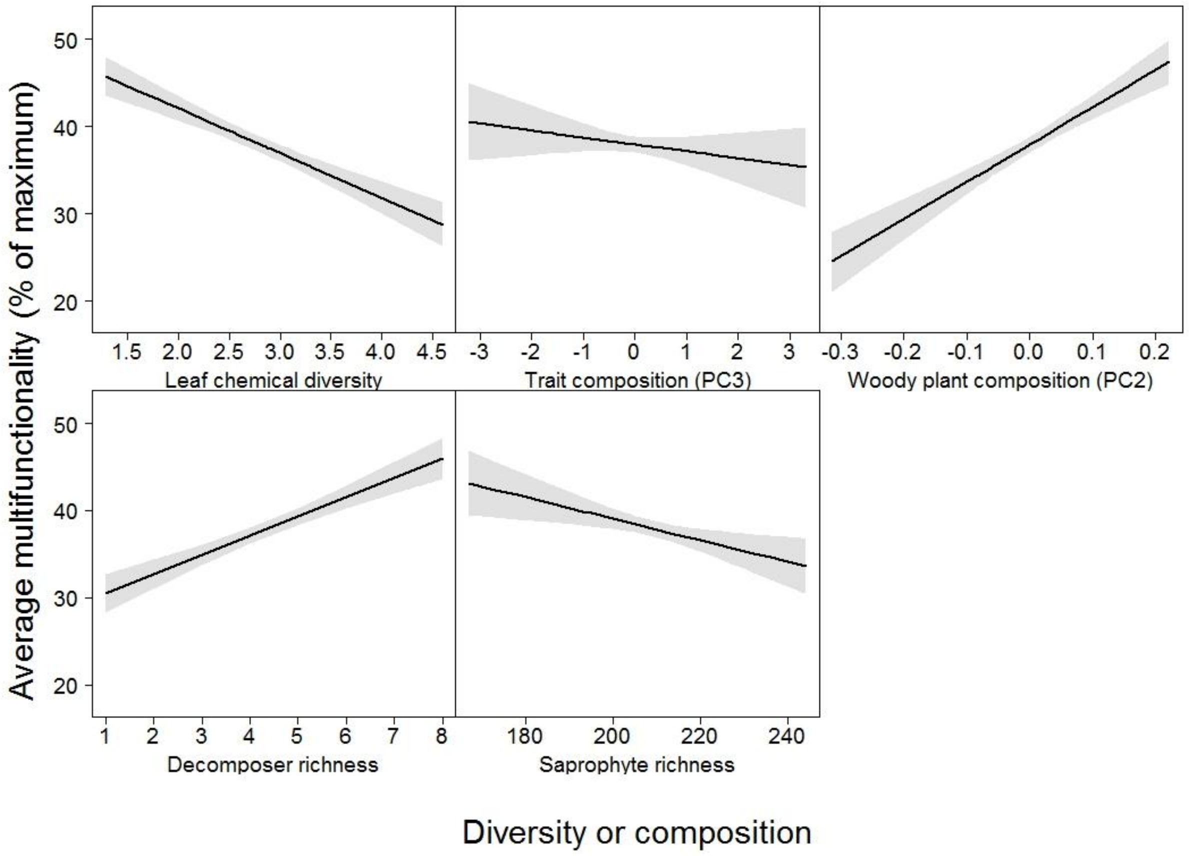

Supplementary Figure 1. Biodiversity effects on average multifunctionality. Effects of woody plant diversity and composition, and heterotrophic species richness (predicted slopes ± 1SE) on multifunctionality as the average of nine standardized ecosystem functions. All regression lines (model predictions) indicate significant ($P \leq 0.05$) relationships. Values on the x-axis represent either increasing diversity (a, d, e) or differences among study plots (n = 26) in species or functional trait composition (b, c).

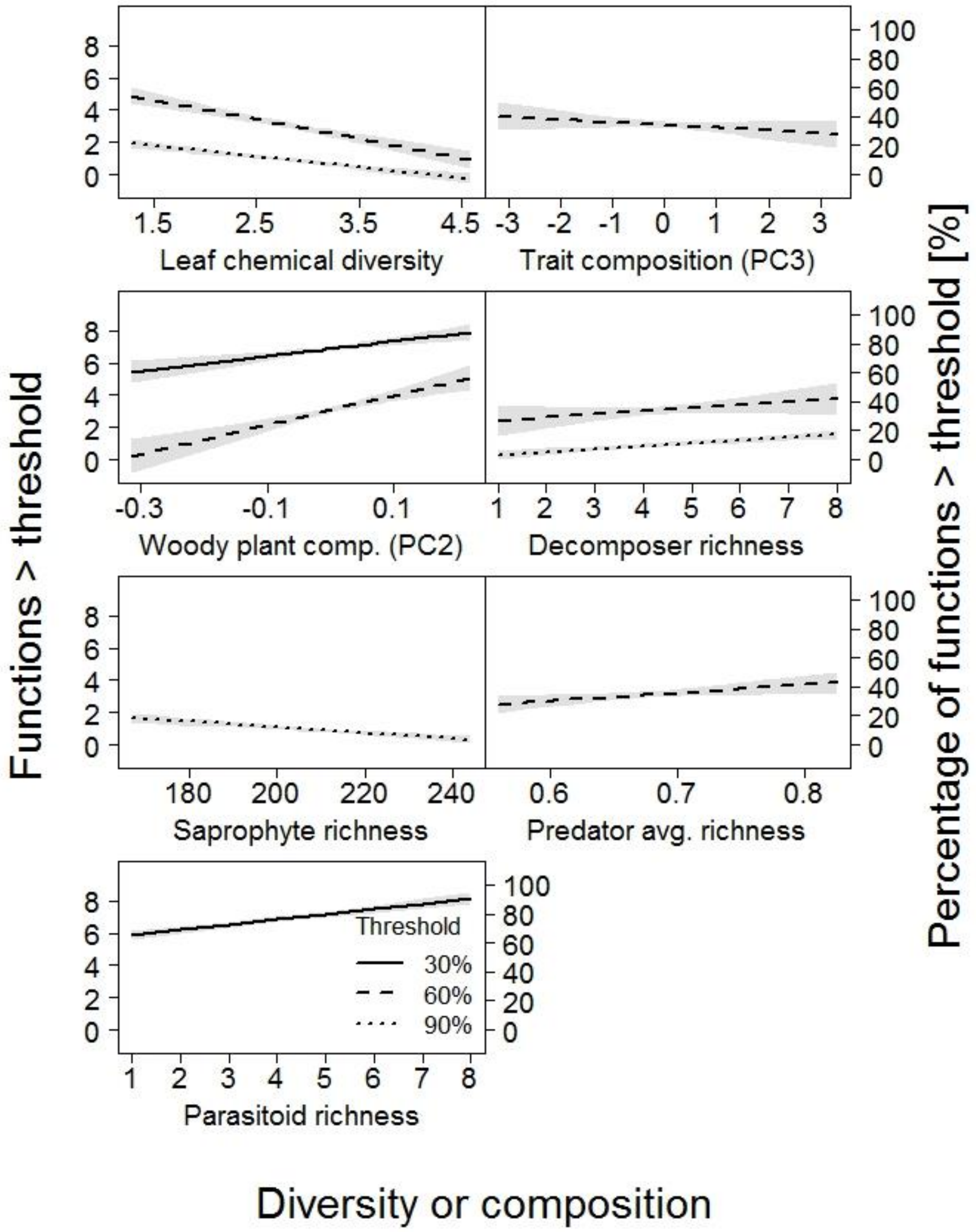

Supplementary Figure 2. Biodiversity effects on multifunctionality thresholds. Effects of woody plant diversity and composition, and heterotrophic species richness (predicted slopes ± 1SE) on multifunctionality as the number of functions surpassing a threshold percentage of the maximum observed value of each function (y-axis labels on the left; axis labels on the right indicate the percentage of the nine functions surpassing the threshold). All regression lines (model predictions) indicate significant ($P \leq 0.05$) relationships. Values on the x-axis represent either increasing diversity or differences among study plots ($n = 26$) in species or functional trait composition.

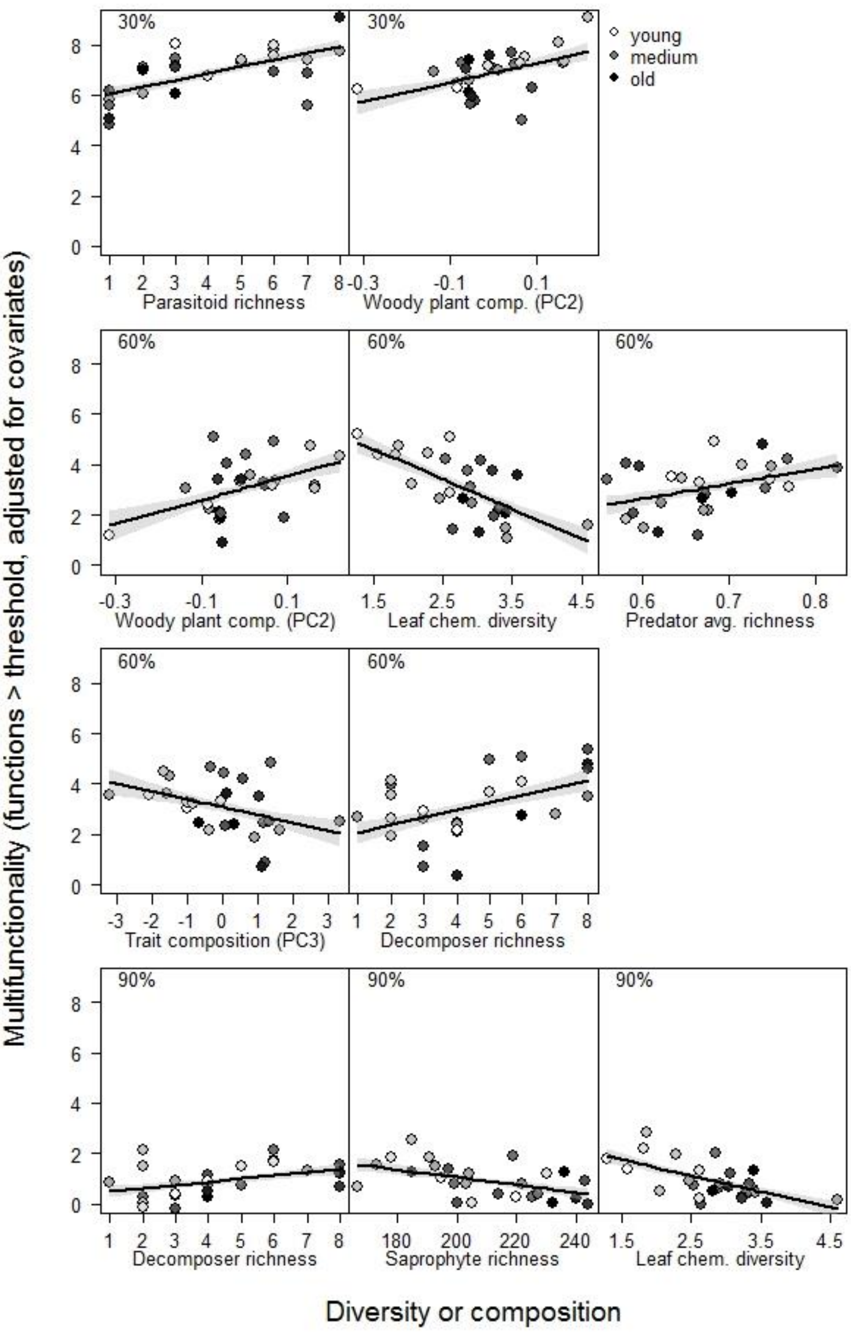

Supplementary Figure 3. Biodiversity effects on multifunctionality thresholds. Effects of woody plant diversity and composition, and heterotrophic species richness on multifunctionality as the number of functions surpassing a threshold percentage (30%, 60%, and 90%) of the maximum observed value of each function. Values on the x-axis represent either increasing diversity or differences among study plots (n = 26) in species or functional trait composition. Note that y-axis values show **data adjusted for covariates** (adding the overall mean of the response variable to residuals obtained after fitting a model including all covariates but not the predictor of interest; see Supplementary Fig. 4 for raw data). Regression lines (± 1SE, fitted across all 26 plots) are adjusted for covariates and indicate significant ($P \leq 0.05$) relationships. The stand age of the study plots is indicated by a continuous gradient from white (youngest plots ~ 20 yr old) to black (oldest plots > 80 yr old).

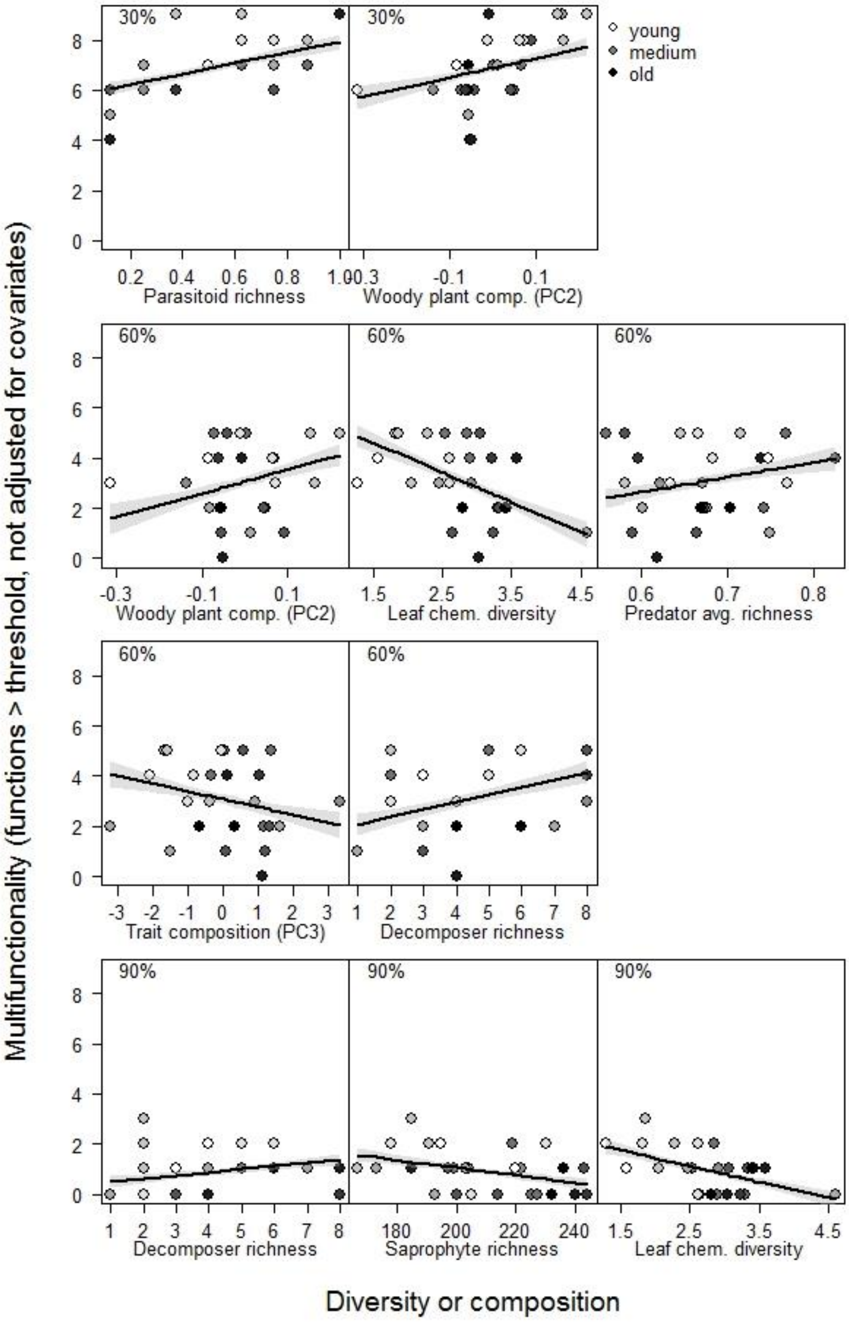

Supplementary Figure 4. Biodiversity effects on multifunctionality thresholds. Effects of woody plant diversity and composition, and heterotrophic species richness on multifunctionality as the number of functions surpassing a threshold percentage (30%, 60%, and 90%) of the maximum observed value of each function. Values on the x-axis represent either increasing diversity or differences among study plots (*n* = 26) in species or functional trait composition. Note that y-axis values show **raw data** (see Supplementary Fig. 3 for adjusted data). Regression lines (± 1SE, fitted across all 26 plots) are adjusted for covariates and indicate significant ($P \leq 0.05$) relationships. The stand age of the study plots is indicated by a continuous gradient from white (youngest plots ~ 20 yr old) to black (oldest plots > 80 yr old).

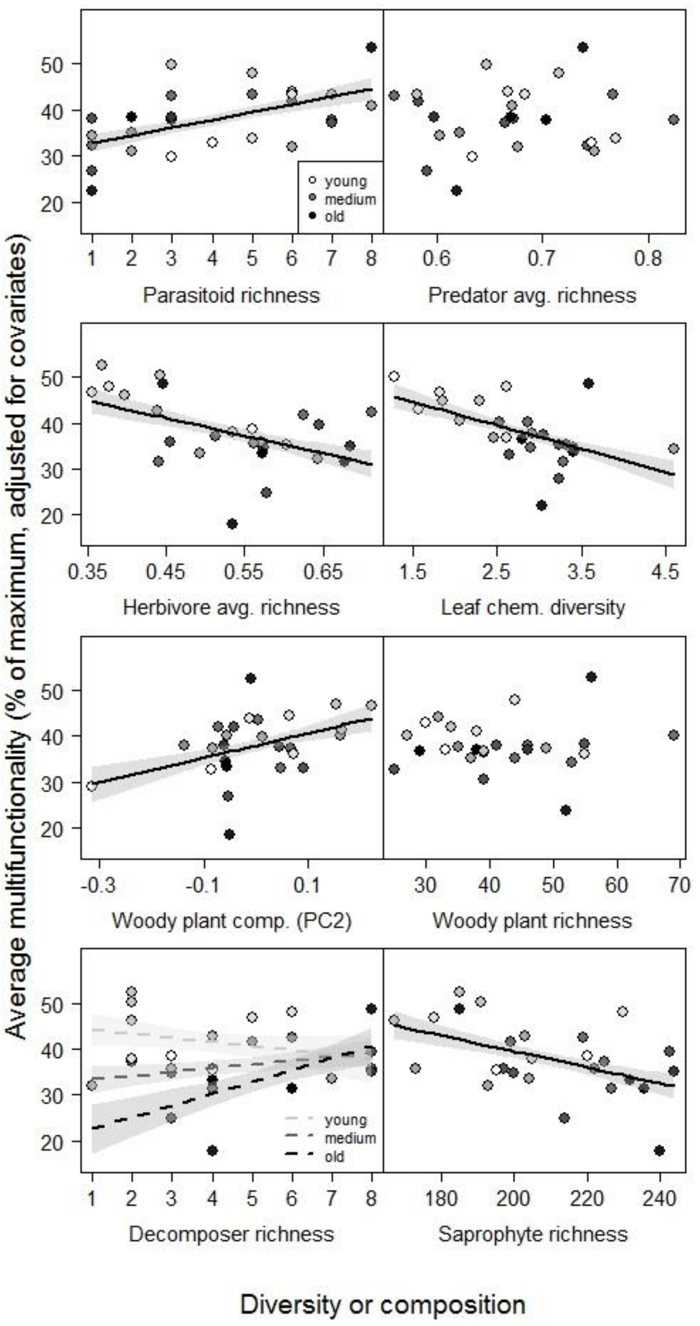

Supplementary Figure 5. Biodiversity effects within individual trophic levels on average multifunctionality. Effects of individual models on woody plant diversity and composition, or heterotrophic species richness of individual trophic levels on multifunctionality as the average of nine standardized ecosystem functions. Values on the x-axis represent either increasing diversity or differences among study plots in species composition. Note that y-axis values show **data adjusted for covariates** (adding the overall mean of the response variable to residuals obtained after fitting a model including all covariates but not the predictor of interest; see Supplementary Fig. 6 for raw data). Solid regression lines (± 1SE, fitted across all 26 plots except for decomposer diversity, where lines are model predictions for young (40 yr), medium (70 yr), and old (100 yr) forest stands) are adjusted for covariates and indicate significant ($P \leq 0.05$) relationships. Broken lines indicate marginally significant ($P < 0.07$) relationships. The stand age of the study plots is indicated by a continuous gradient from white (youngest plots ~ 20 yr old) to black (oldest plots > 80 yr old); avg. = average.

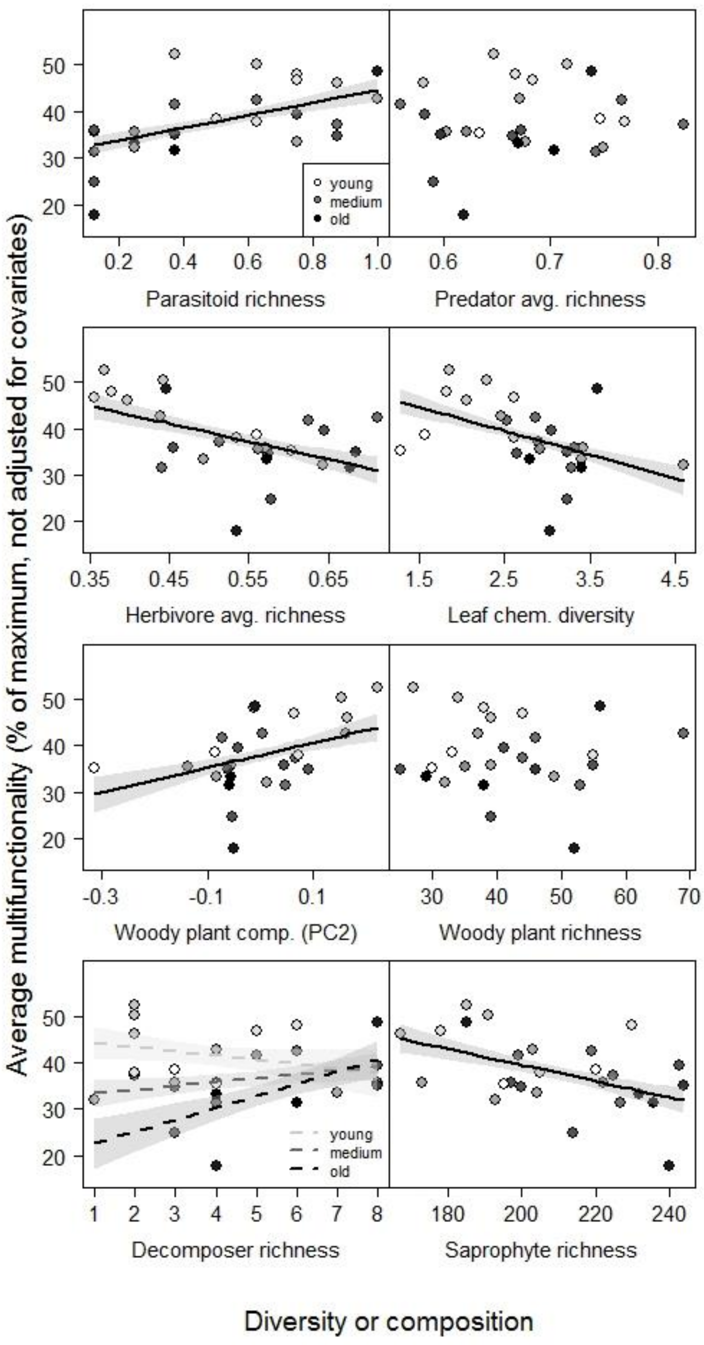

Supplementary Figure 6. Biodiversity effects within individual trophic levels on average multifunctionality. Effects of individual models on woody plant diversity and composition, or heterotrophic species richness of individual trophic levels on multifunctionality as the average of nine standardized ecosystem functions. Values on the x-axis represent either increasing diversity or differences among study plots in species composition. Note that y-axis values show **raw data** (see Supplementary Fig. 5 for adjusted data). Solid regression lines (± 1SE, fitted across all 26 plots except for decomposer diversity, where lines are model predictions for young (40 yr), medium (70 yr), and old (100 yr) forest stands) are adjusted for covariates and indicate significant ($P \leq 0.05$) relationships. Broken lines indicate marginally significant ($P < 0.07$) relationships. The stand age of the study plots is indicated by a continuous gradient from white (youngest plots ~ 20 yr old) to black (oldest plots > 80 yr old); avg. = average.

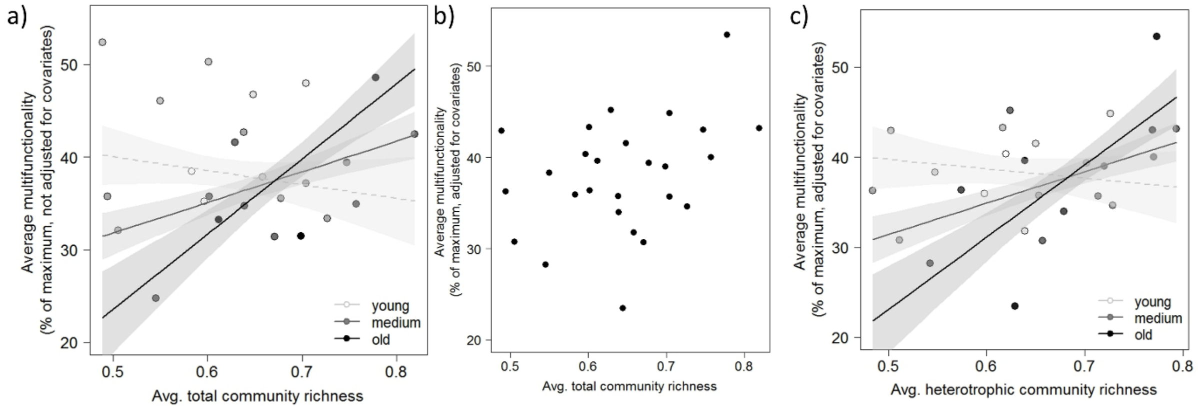

Supplementary Figure 7. Overall multidiversity effects on average multifunctionality. Effects of a & b) average total community richness (average of standardized species richness across trophic levels, including tree species richness; panel b shows the overall relationship irrespective of stand age of the study plots) and c) heterotrophic average community richness (excluding tree species richness) on multifunctionality as the average of nine standardized ecosystem functions. Note that y-axis values in a) show **raw data** (see Figure 4 for data adjusted for covariates), values in b) and c) are **data adjusted for covariates** (adding the overall mean of the response variable to residuals obtained after fitting a model including all covariates but not the predictor of interest). Solid regression lines (model predictions for young (40 yr), medium (70 yr), and old (100 yr) forest stands) are adjusted for covariables and indicate significant ($P \leq 0.05$) relationships. The broken line indicates non-significant relationships. The stand age of the study plots (n = 26) is indicated by a continuous gradient from white (youngest plots ~ 20 yr old) to black (oldest plots > 80 yr old); avg. = average.

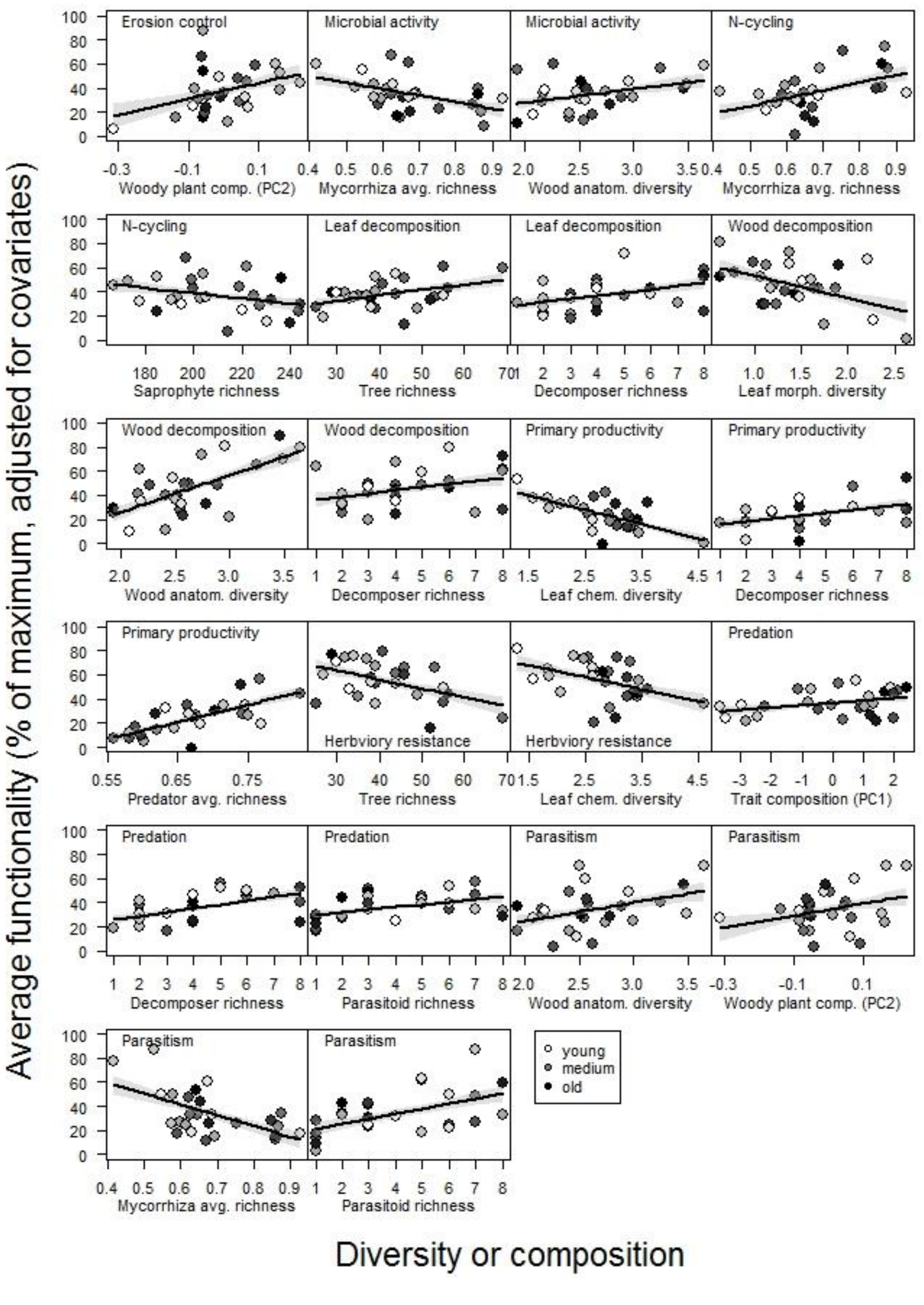

Supplementary Figure 8. Biodiversity effects on individual ecosystem functions. Effects of woody plant diversity and composition, and heterotrophic species richness. Values on the x-axis represent either increasing diversity or differences among study plots in species or functional trait composition. Note that y-axis values show **data adjusted for covariates** (adding the overall mean of the response variable to residuals obtained after fitting a model including all covariates but not the predictor of interest; see Supplementary Fig. 9 for raw data). Regression lines (± 1SE, fitted across all 26 plots) are adjusted for covariates and indicate significant ($P \leq 0.05$) relationships. The stand age of the study plots is indicated by a continuous gradient from white (youngest plots ~ 20 yr old) to black (oldest plots > 80 yr old).

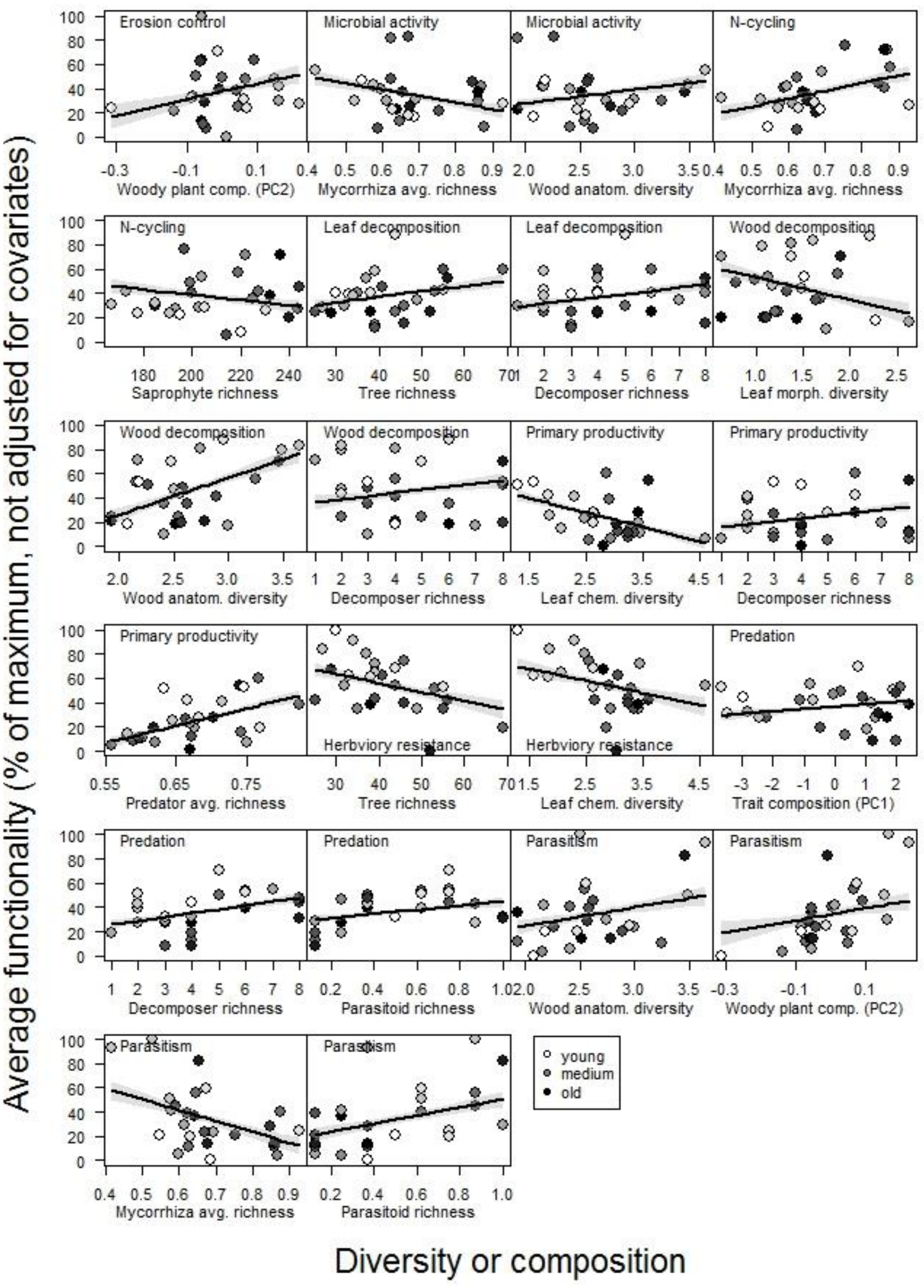

Supplementary Figure 9. Biodiversity effects on individual ecosystem functions. Effects of woody plant diversity and composition, and heterotrophic species richness. Values on the x-axis represent either increasing diversity or differences among study plots in species or functional trait composition. Note that y-axis values show **raw data** (see Supplementary Fig. 8 for adjusted data). Regression lines (± 1SE, fitted across all 26 plots) are adjusted for covariates and indicate significant ($P \leq 0.05$) relationships. The stand age of the study plots is indicated by a continuous gradient from white (youngest plots ~ 20 yr old) to black (oldest plots > 80 yr old).

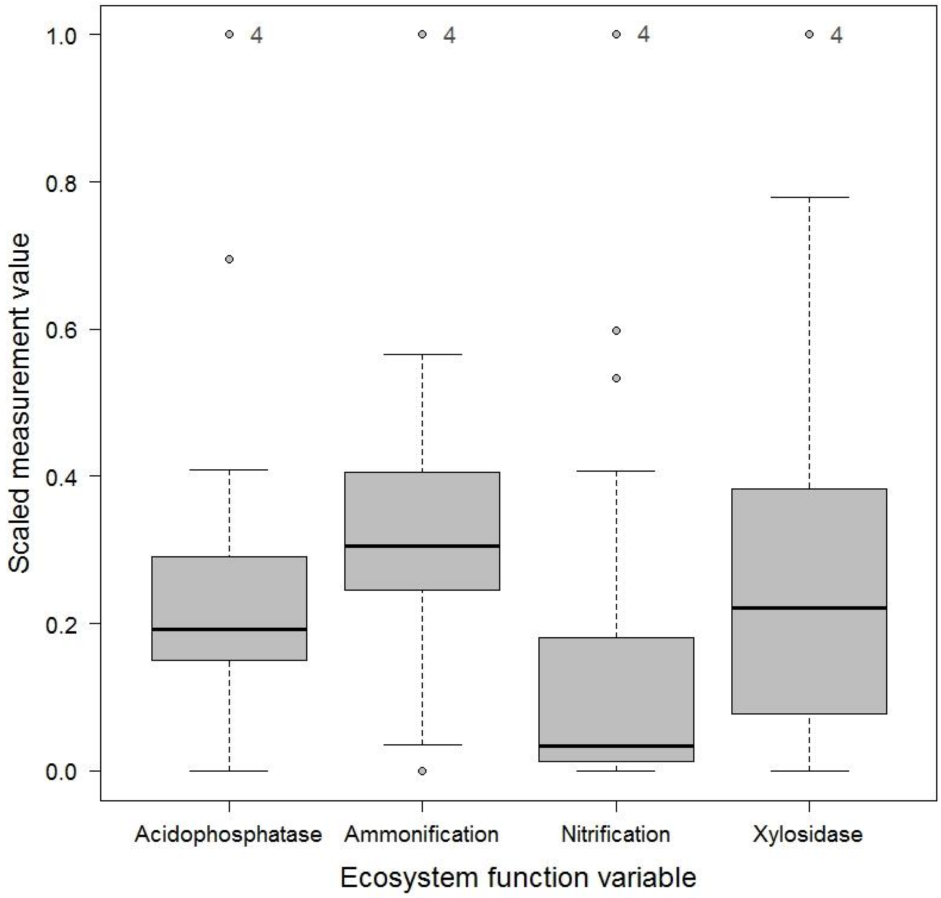

Supplementary Figure 10. Distribution of measurement values (scaled between 0 and 1) of four soil-based ecosystem function measurements. Study plot 4 (outlier points at the top of the graph) was excluded from the multifunctionality analyses because the extreme values suggested processing errors of the soil samples of this study plot.

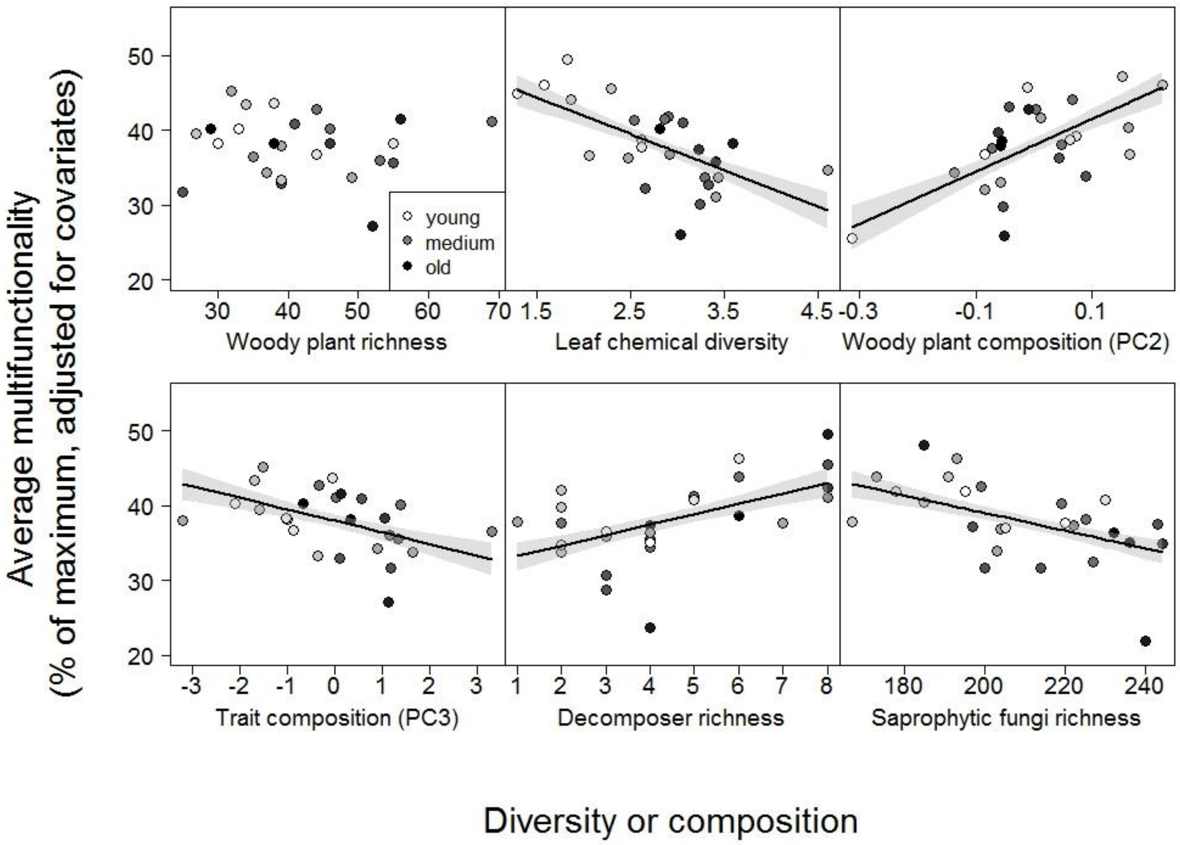

Supplementary Figure 11. Biodiversity effects on average multifunctionality. Multimodel-averaging results for effects of woody plant diversity and composition, and heterotrophic species richness on multifunctionality as the average of nine standardized ecosystem functions in a biodiverse subtropical forest. Only variables retained after model simplification and model averaging (as well as tree species richness for comparison) are shown. Values on the x-axis represent either increasing diversity or differences among study plots in species or functional trait composition. Note that y-axis values show **raw data** (see Figure 1 for data adjusted for covariates). Regression lines (± 1SE, fitted across all 26 plots) are adjusted for covariates and indicate significant ($P \leq 0.05$) relationships. The stand age of the study plots is indicated by a continuous gradient from white (youngest plots ~ 20 yr old) to black (oldest plots > 80 yr old).

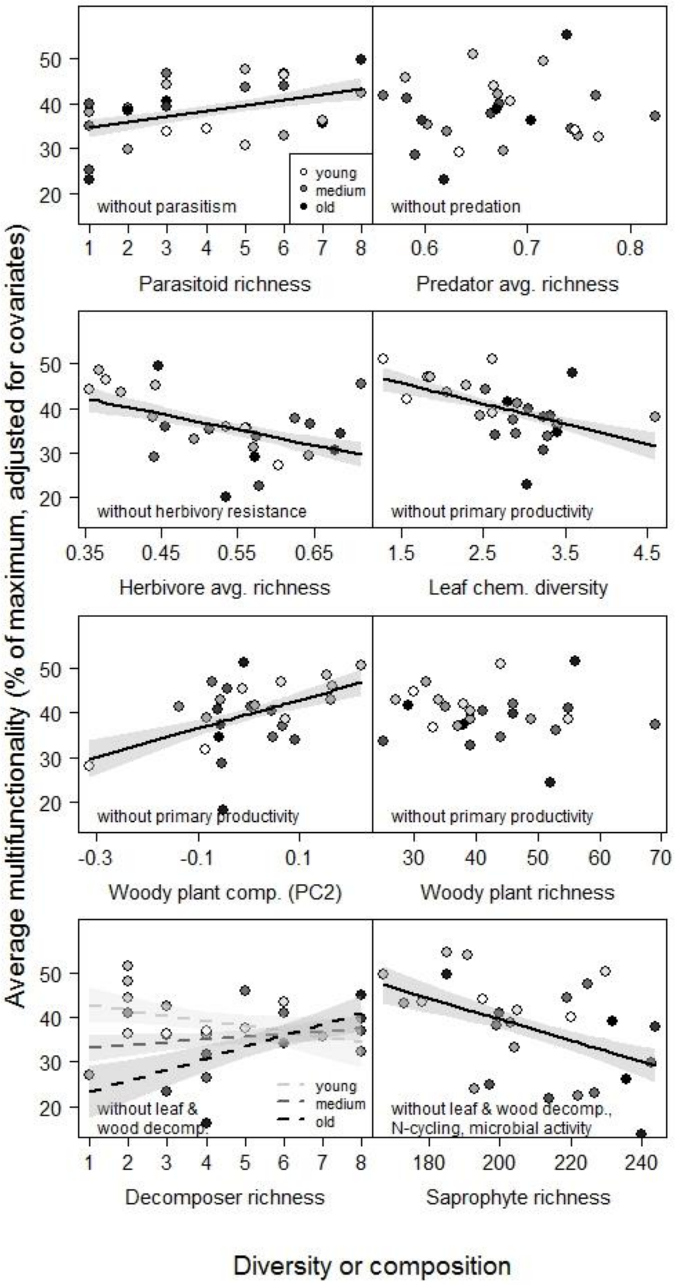

Supplementary Figure 12. Biodiversity effects within individual trophic levels on average multifunctionality. Effects of individual models on woody plant diversity and composition, or heterotrophic species richness of individual trophic levels on multifunctionality as the average of five to eight standardized ecosystem functions (excluding functions directly mediated by a given trophic level). Values on the x-axis represent either increasing diversity or differences among study plots in species composition. Note that y-axis values show **raw data** (see Figure 3 for data adjusted for covariates). Solid regression lines (± 1SE, fitted across all 26 plots except for decomposer diversity, where lines are model predictions for young (40 yr), medium (70 yr), and old (100 yr) forest stands) are adjusted for covariates and indicate significant ($P \leq 0.05$) relationships. Broken lines indicate marginally significant ($P < 0.07$) relationships.. The stand age of the study plots is indicated by a continuous gradient from white (youngest plots ~ 20 yr old) to black (oldest plots > 80 yr old); avg. = average.

Supplementary References

1 Pei, Z. et al. Leaf litter diversity alters microbial activity, microbial abundances, and nutrient cycling in a subtropical forest ecosystem. *Biogeochemistry* **134**, 163-181 (2017).

2 Oates, L. G. et al. A lipid extraction and analysis method for characterizing soil microbes in experiments with many samples. *J. Visual. Exp.*, 55310 (2017).

3 Trogisch, S. et al. Toward a methodical framework for comprehensively assessing forest multifunctionality. *Ecol. Evol.* **7**, 10652–10674, doi: 10.1002/ece1003.3488 (2017).

4 De Boer, W., Hundscheid, M. P. J., Schotman, J. M. T., Troelstra, S. R. & Laanbroek, H. J. In situ net N transformations in pine, fir, and oak stands of different ages on acid sandy soil, 3 years after liming. *Biol. Fertil. Soil* **15**, 120-126 (1993).

5 Hart, S., Stark, J., Davidson, E. & Firestone, M. K. in Methods of soil analysis. Part 2: Microbial and bio-chemical properties (eds R. Weaver et al.) 985-1018 (Soil Science Society of America, 1994).

6 Brezzi, M. Influence of tree species richness on arthropod community patterns and foliar herbivory plus allometric equations to predict tree biomass, University of Zurich, (2015).

7 Olson, J. S. Energy storage and the balance of producers and decomposers in ecological systems. Ecology 44, 322-331 (1963).

8 Geißler, C. et al. Kinetic energy of throughfall in subtropical forests of SE China ? Effects of tree canopy structure, functional traits, and biodiversity. *PLoS ONE* **8**, e49618 (2013).

9 Pei, Z. et al. Soil and tree species traits both shape soil microbial communities during early growth of Chinese subtropical forests. *Soil Biol. Biochem.* **96**, 180-190 (2016).

10 Trogisch, S., He, J.-S., Hector, A. & Scherer-Lorenzen, M. Impact of species diversity, stand age and environmental factors on leaf litter decomposition in subtropical forests in China. *Plant Soil* **400**, 337-350 (2016).

11 Baruffol, M. et al. Biodiversity promotes tree growth during succession in subtropical forest. *PLoS ONE* **8**, e81246 (2013).

12 Brezzi, M., Schmid, B., Niklaus, P. A. & Schuldt, A. Tree diversity increases levels of herbivore damage in a subtropical forest canopy: evidence for dietary mixing by arthropods? *J. Plant Ecol.* **10**, 13-27 (2017).

13 Schuldt, A. et al. Tree diversity promotes insect herbivory in subtropical forests of south-east China. J. Ecol. 98, 917-926 (2010).

14 Schuldt, A. & Staab, M. Tree species richness strengthens relationships between ants and the functional composition of spider assemblages in a highly diverse forest. *Biotropica* **47**, 339-346 (2015).

15 Staab, M. et al. Tree phylogenetic diversity promotes host–parasitoid interactions. *Proc. R. Soc. B* **283**, 20160275 (2016).

16 Binkenstein, J. *et al.* Multi-trophic guilds respond differently to changing elevation in a subtropical forest. *Ecography*, doi: 10.1111/ecog.03086 (2018).

17 Wu, Y. T. *et al.* Forest age and plant species composition determine the soil fungal community composition in a Chinese subtropical forest. *PLoS ONE* **8**, e66829 (2013).

18 Gao, C. *et al.* Community assembly of ectomycorrhizal fungi along a subtropical secondary forest succession. *New Phytol.* **205**, 771-785 (2015).

19 Schuldt, A. *et al.* Woody plant phylogenetic diversity mediates bottom-up control of arthropod biomass in species-rich forests. *Oecologia* **176**, 171-182 (2014).

20 O'Brien, M. J. *et al.* Tree diversity drives diversity of arthropod herbivores, but successional stage mediates detritivores. *Ecol. Evol.* **7**, 8753-8760 (2017).
21 Schuldt, A. *et al.* Predator diversity and abundance provide little support for the enemies hypothesis in forests of high tree diversity. *PLoS ONE* **6**, e22905 (2011).
22 Schuldt, A. *et al.* Tree diversity promotes functional dissimilarity and maintains functional richness despite species loss in predator assemblages. *Oecologia* **174**, 533-543 (2014).
23 Schuldt, A., Bruelheide, H., Härdtle, W. & Assmann, T. Predator assemblage structure and temporal variability of species richness and abundance in forests of high tree diversity. *Biotropica* **44**, 793-800 (2012).
24 Staab, M., Schuldt, A., Assmann, T., Bruelheide, H. & Klein, A.-M. Ant community structure during forest succession in a subtropical forest in South-East China. *Acta Oecol.* **61**, 32-40 (2014).
25 Staab, M., Schuldt, A., Assmann, T. & Klein, A. M. Tree diversity promotes predator but not omnivore ants in a subtropical Chinese forest. *Ecol. Entomol.* **39**, 637-647 (2014).
26 Schuldt, A. *et al.* Multitrophic diversity in a biodiverse forest is highly nonlinear across spatial scales. *Nat. Commun.* **6**, 10169; doi: 10110.11038/ncomms10169 (2015).
27 Schuldt, A. *et al.* Belowground top-down and aboveground bottom-up effects structure multitrophic community relationships in a biodiverse forest. *Sci. Rep.* **7**, 4222 (2017).
28 Wu, Y. T. *et al.* Relationships between soil microorganisms, plant communities, and soil characteristics in Chinese subtropical forests. *Ecosystems* **15**, 624-636 (2012).
29 Eichenberg, D., Trogisch, S., Huang, Y., He, J.-S. & Bruelheide, H. Shifts in community leaf functional traits are related to litter decomposition along a secondary forest succession series in subtropical China. *J. Plant Ecol.* **8**, 401-410 (2015).
30 Castro-Izaguirre, N. *et al.* Tree diversity enhances stand carbon storage but not leaf area in a subtropical forest. *PLoS ONE* **11**, e0167771 (2016).
31 Schuldt, A. *et al.* Functional and phylogenetic diversity of woody plants drive herbivory in a highly diverse forest. *New Phytol.* **202**, 864-873 (2014).
32 Purahong, W. *et al.* Characterization of unexplored deadwood mycobiome in highly diverse subtropical forests using culture-independent molecular technique. *Front. Microbiol.* **8**, 574 (2017).
